# Morphological and environmental varieties of the Cambrian stromatolites in Western Henan, North China Craton

**DOI:** 10.1371/journal.pone.0319713

**Published:** 2025-03-28

**Authors:** Yu-Guang Chang, Shi-Yun Qing, Ming-Yue Dai, Song-Hua Chen

**Affiliations:** 1 Henan Polytechnic University, Jiaozuo, Henan, China; 2 Henan International Joint Laboratory of Biogenic Traces and Sedimentary Minerals, Jiaozuo, Henan, China; Birbal Sahni Institute of Palaeosciences: Birbal Sahni Institute of Palaeobotany, INDIA

## Abstract

Morphological varieties of Cambrian stromatolites in western Henan, North China Craton were essentially related to palaeoenvironmental parameter changes of the marine-floor. Small plexiform, hemispherical-smooth wavy, small columnar, irregular small columnar stromatolites, and stromatolites that surrounding flat-pebble conglomerate limestones are identified, in which the last type of stromatolite is unique, and they possess different macromorphology and micromorphology characteristics. A large number of *Girvanella* fossils, which were distributed in prostrate or horizontal and winding or overlapping patterns, were preserved in the laminae of stromatolites, and the dark laminae were formed by combined effect of *Girvanella* and precipitation of calcium carbonate. The research elucidates that small plexiform and small columnar stromatolite developed in the supratidal zone, hemispherical-smooth wavy, small columnar stromatolites, and stromatolites that surrounding flat-pebble conglomerate limestones grew in the intertidal zone with weaker hydrodynamic conditions, as well as irregular small columnar stromatolites occurred in subtidal zone. The features of microscopic laminae of stromatolites are influenced by microorganisms and metazoan, the macroscopic morphologies and growth of stromatolites are affected and controlled by terrigenous materials, hydrodynamic conditions and sedimentary substrates.

## 1. Introduction

**Fig 1:**
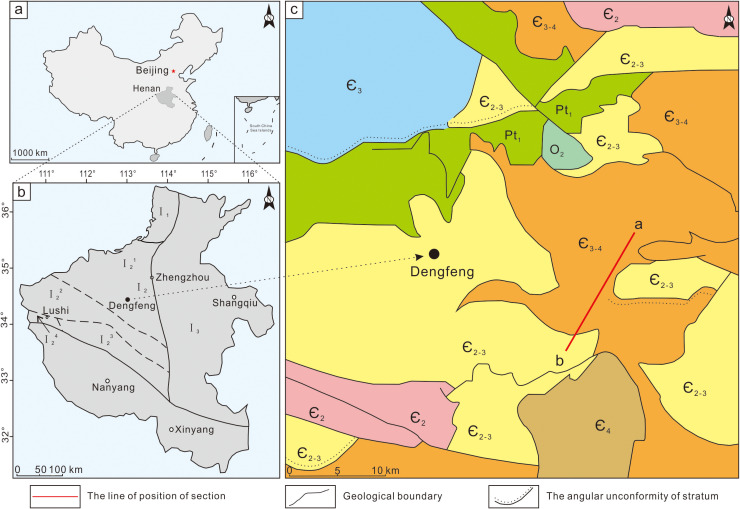
Geological map showing the Cambrian stratigraphic distribution and section localities in western Henan. Reprinted from Liu et al under a CC BY license, with permission from Geological Publishing House, Beijing, China, original copyright 1991 [21]. (a, b) Guankou section is located in western Henan, China. (c) Cambrian System of Dengfeng area: a-b, Guankou section.

Stromatolites, which have occupied the earth for more than 3 billion years [1–4] and have preserved a great deal of information about microorganisms, environment, geochemistry and geophysics, etc [5–7], are a significant sedimentary record in the evolution of the earth [8]. However, stromatolites have declined since the Phanerozoic due to the emergence of metazoans and changes in environment[9]. Cambrian microbialites formed by *Girvanella* and other microorganisms together with metazoan consorters widely distributed in carbonate platform [10,11]. Columnar stromatolitic reefs[12,13], stromatolite-rimmed thrombolite columns and domes constructed by microstromatolites, calcimicrobes and sponges in USA[14,15], pleomorphic thrombolite-stromatolite complex communities in Australia [16], wavy and columnar stromatolites in North China Craton [11] are of the diverse microbialitic cases. Ecological interpretations on stromatolitic microorganisms and sedimentary environments were primarily literatured [4,17–20]. Further investigation on visible microbial fabrics should be directly demonstrate biotic structures of the stromatolites. Lithological loggings on Cambrian sequences from Guankou section in western Henan [21] (Fig 1a, 1b) was described as carbonates successed by siliciclasts [22,23]; stromatolites are well outcropped with profuse types and distinctive macromorphology. On the basis of analysis of micromorphology and micromorphology of stromatolites, controlling factors on stromatolitic morphology are probed. We provide a basis for clarifying the microbial genesis and the sharp attenuation of the stromatolites, and paleoenvironmental and paleoclimatic reconstructions.

## 2. Morphology of stromatolites

**Fig 2:**
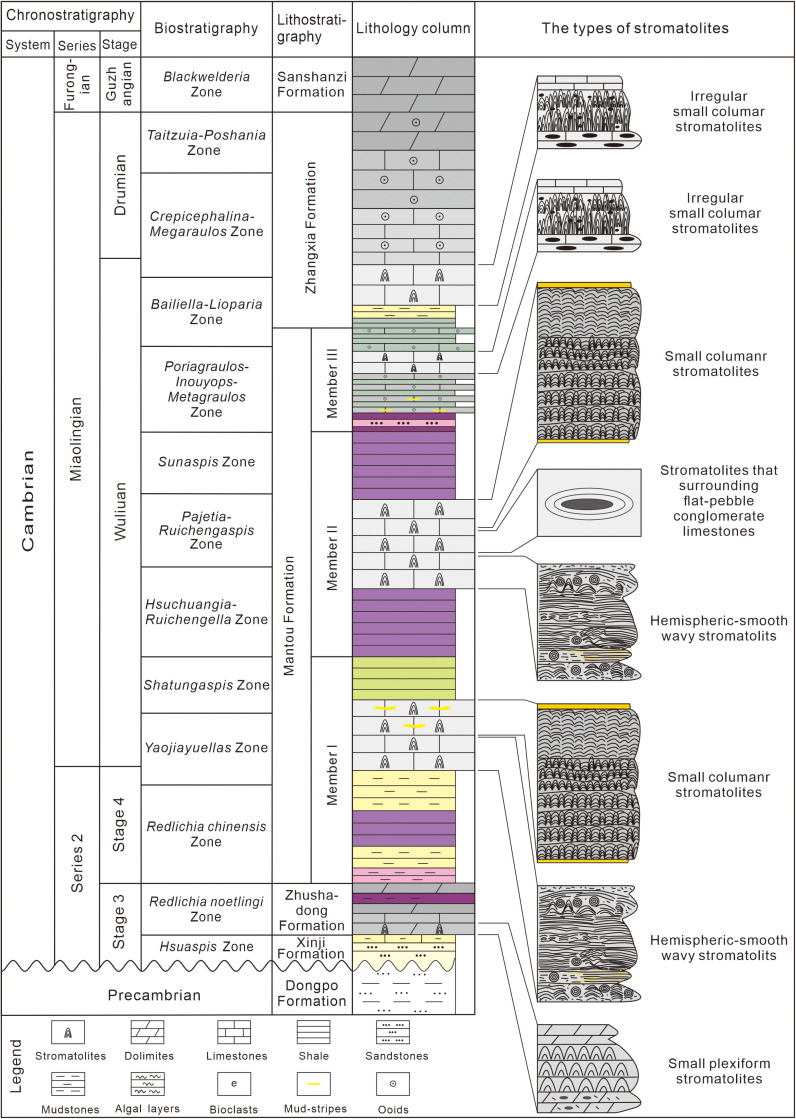
Lithostratigraphy and the types of stromatolites of the Cambrian in Guankou section. Figure modified from Pei et al. [24].

Cambrian stromatolites vertically occur from Zhushadong to Zhangxia Formations (Fig 2).

**Fig 3:**
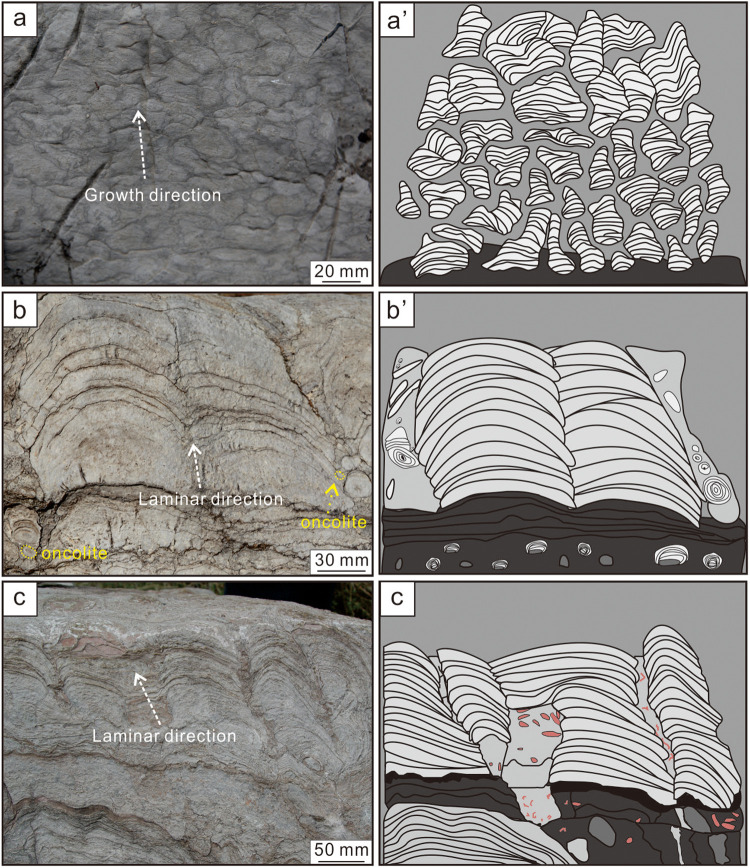
Field details of stromatolites. (a) Macromorphology of small plexiform stromatolites in Zhushadong Formation; (b) Macromorphology of hemispheric stromatolites in Mantou Formation; (c) Macromorphology of wavy smooth stromatolites in Mantou Formation; (a’-c’) Sketches of corresponding stromatolites.

**Fig 4:**
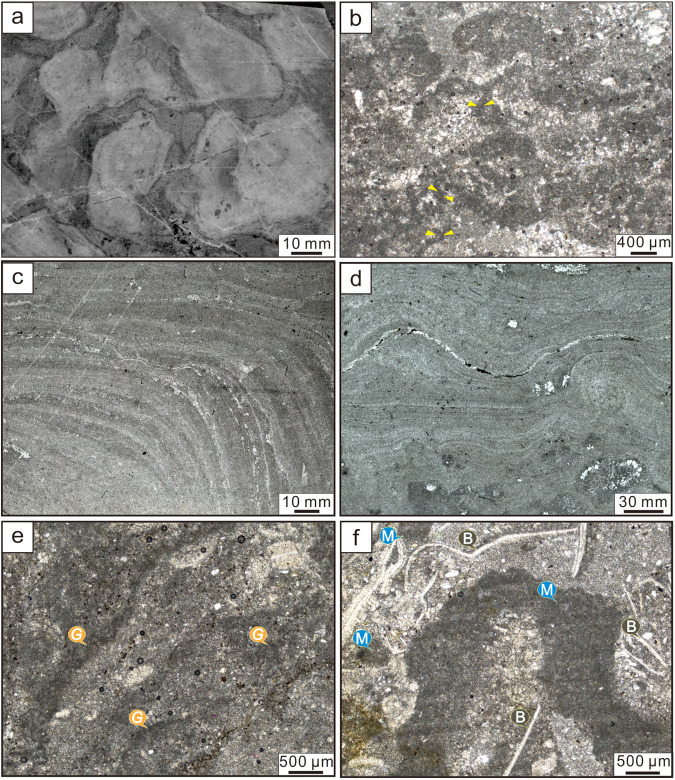
Micrographs of stromatolites. (a, b) Micromorphology of small plexiform stromatolites; (c) Micromorphology of hemispheric stromatolites; (d) Micromorphology of wavy smooth stromatolites; (e) Micromorphology of small columnar stromatolites; (f) Micromorphology of irregular small columnar stromatolites. The symbol G points to *Girvanella* filaments, M points to the *Girvanella* masses, B points to the biodetritus.

Small plexiform stromatolites show irregular macroscopic morphologies such as plexiform or digitate and present slight branching. Diameter individual column ranges 1-3 cm. Dense plexiform stromatolites are upwardly narrowed and sub-rounded, rounded or irregular in plan view. The fillings between stromatolites show distinctly square-mesh (Fig 3a, 3a’). Fine laminae are arched or cloudy form (Fig 4a). Dark organic matters are observed in cracks of microcrystalline calcites and calcite, sparry calcites with more than 200 µm occasionally (Fig 4b).

Hemispheric stromatolites are abundant in the Cambrian Stage 4 and Wuliuan Stage (Fig 2). Hemispheric and smooth wavy stromatolites are commonly co-existance in a same bed. The hemispheric parts display regular concentric circles in plan view and hemispheric shape in sectional view (Fig 3b, 3b’), mostly size as 5-20 cm in diameter and 5-15 cm in high. Smooth wavy stromatolites generally exposed in the upper and lower layers of columnar stromatolites (Fig 2), ca 5-20 cm in diameter. The laminae of stromatolites undulate to a certain extent and their bedding boundaries are generally visible (Fig 3c, 3c’).

The hemispheric stromatolites contain a small amount of shell fossils. The dark laminae consist of micritic calcites with small and uniform particles size, while the light laminae are composed of microsparry calcites or sparry calcites with large particle size (Fig 4c). The light and dark laminae of smooth wavy stromatolites are relatively conspicuous in the polarizing microscope. The dark laminae are thicker than light laminae (Fig 4d).

**Fig 5:**
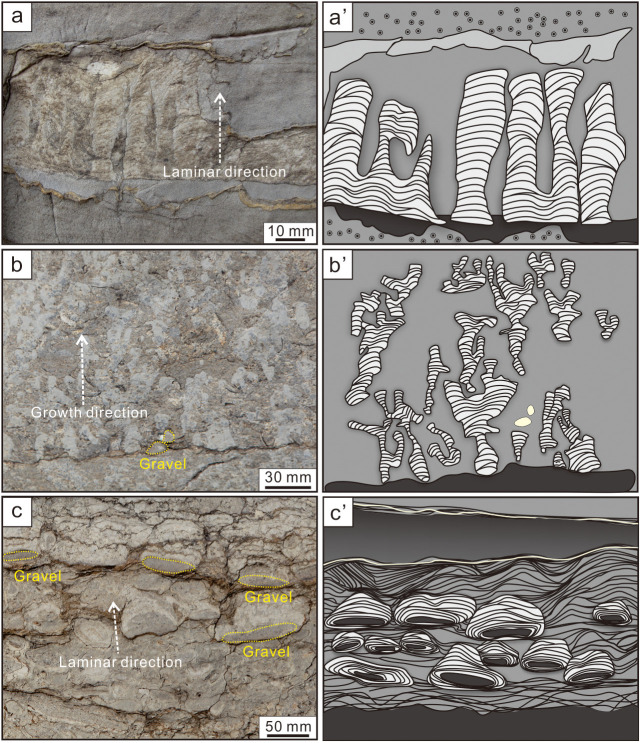
Field details of stromatolites. (a) Macromorphology of small columnar stromatolites in Mantou Formation; (b) Macromorphology of irregular small columnar stromatolites in Zhangxia Formation; (c) Macromorphology of stromatolites that surrounding flat-pebble conglomerate limestones in Mantou Formation; (a’-c’) Sketches of corresponding stromatolites.

Small columnar stromatolites are multifarious morphologies such as micro columnar, digitate and without branching. The individual column is about 2-3 cm in diameter and 2-4 cm high (Fig 5a, 5a’). They are often interrupted by thin-bedded mudstones and upwardly growth to form columnar or smooth wavy stromatolites. The light laminae consist of sparry calcites with larger particle size, while the dark laminae are made up of sparry calcites with finer particle size and many microbial filaments (Fig 4e).

Irregular small columnar stromatolites show irregular morphologies such as bundle-like, small plexiform, digitate and branching, size as 1-3 cm in diameter and 2-3 cm high (Fig 5b, 5b’). Distinct boundaries between top and bottom. A large number of filamentous microfossils are detected in dark laminae, usually prostrate, occasionally vertical distribution. When they are accompanied by uniform and dense biodetritus such as trilobites, present obviously granular distribution with uneven particle size, and grow on the larger biodetritus to form sheet-mat (Fig 4f).

Stromatolites that surrounding flat-pebble conglomerate limestones described herein are unique and have been almost no relevant report before. The flat-pebble gravels are mainly marls, generally 5-12 cm in length, with a certain degree of roundness and horizontal distribution. The morphology of stromatolites is influenced by the shape of flat-pebble gravels, show oval in plan view, and slightly pointed at both ends (Fig 5c, 5c’).

## 3. Microbial structures

**Fig 6:**
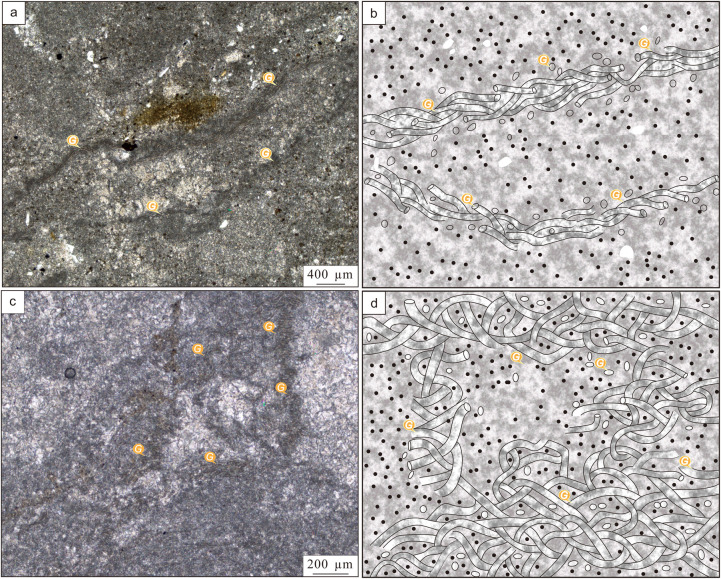
The characteristics and distribution of *Girvanella* in the laminae of stromatolites. (a) *Girvanella* filaments are distributed in the dark laminae of small columnar stromatolites, arranged in prostrate and horizontal form, or intertwined into ropes. (b) Sketch of distribution form of *Girvanella* in b. (c) *Girvanella* filaments are distributed in the dark laminae of irregular small columnar stromatolites, arranged in the form of intertwining or overlapping. (d) Sketch of distribution form of *Girvanella* in d. The symbol *G* points to *Girvanella*.

Filamentous microfossils appear as elongated, slightly twisted, unbranched calcareous tubular texture, uniform diameter and extension length of more than 200 µm, and commonly intertwine into mesh-like masses or ropes. They are elliptical to circular in cross section, cylindrical in longitudinal section, and consist of tube core and tube wall, in which tube core is 5-9 µm in diameter and composed of microsparry calcites, while tube wall is 1-2 µm in thickness and made up of micritic calcites (Figs 4e and 6). The characteristics indicate that filamentous microfossils conform to the definition proposed by Nicholoson & Ethridge [25], and belong to the typical *Girvanella*.

*Girvanella* filaments are distributed in the dark laminae in two patterns: (1) Prostrate or horizontal distribution. *Girvanella* filaments are prostrate or approximately horizontally arranged, or intertwine into ropes. The dark laminae are eventually formed by combined action of *Girvanella* and precipitation of calcium carbonate. Occasionally, they can be observed growing between bioclasts or ooids (Fig 6a, 6b), indicating that hydrodynamic conditions are higher when they are formed. (2) Winding or overlapping distribution. *Girvanella* filaments intertwine with each other and overlapping outwards along the laminae in prostrate, horizontal or inclined manners, with no obvious growth direction and forming thicker dark lamina (Fig 6c, 6d).

## 4. Analysis of sedimentary environments of stromatolites

The sedimentary environment of stromatolites can be inferred by analysis of macromorphology and micromorphology characteristics of stromatolites and combining with sedimentary environments of modern and Precambrian stromatolites [9,26,27].

### 4.1. Small plexiform stromatolites

**Fig 7:**
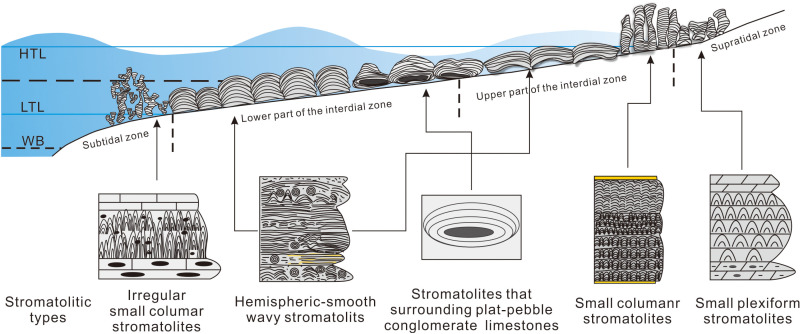
The distribution of sedimentary environment of stromatolites. Stromatolites are relatively concentrated in the intertidal zone, and a small amount are distributed in the supratidal zone and subtidal zone.

Small plexiform stromatolites, with dark grey dolomites containing chert lumps and small gravels as the bottom, and microcrystalline or laminated dolomites as the top, expose in light grey dolomites (Figs 2 and 3a). Taking microsparry calcites with smaller particle size as the matrix, sparry calcites with larger particle size can be seen occasionally, and no bioclastics are found (Fig 4a, 4b), reflecting that the hydrodynamic conditions are weaker. Therefore, it is can be judged that small plexiform stromatolites grow in supratidal zone with shallow water and weaker hydrodynamic conditions (Fig 7).

### 4.2. Hemispheric-smooth wavy stromatolites

Hemispherical stromatolites mainly develop in light gray limestones or marls. A large number of flat-pebble gravels, oncolites and biodetritus are found in surrounding rocks. The intervals of stromatolites are filled with oncolites and biodetritus with higher degree of fragmentation (Fig 3b). The sparry calcites with larger particle size and the biodetritus can be discovered in light laminae (Fig 4c). Therefore, it can be inferred that hemispherical stromatolites grow in the lower part of the intertidal zone with higher energy environment (Fig 7).

Smooth wavy stromatolites occur in limestones or marls and generally expose in upper and lower layers of small columnar stromatolites, and are formed by the fusion of the latter interrupted by thin-bedded mudstones. The flat-pebble gravels or mud-stripes can be observed in surrounding rocks (Fig 3c), as well as hemispherical stromatolites associated with smooth wavy stromatolites. It reveals that smooth wavy stromatolites primarily formed in the upper part of intertidal zone and the lower part of supratidal zone (Fig 7).

### 4.3. Small columnar stromatolites

Small columnar stromatolites grow in marls or limestones containing muds or ooids, with mudstones or algal layers at the top. The intervals of stromatolites are filled with muds, ooids and bioclastics (Figs 5a and 6a). The particle size is uniform and fine, and *Girvanella* fossils and biodetritus with lower degree of fragmentation can be seen (Figs 4e and 6a, 6b). It reflects that small columnar develop in the upper part of intertidal zone with weaker hydrodynamic conditions or the lower part of supratidal zone (Fig 7).

### 4.4. Irregular small columnar stromatolites

Irregular small columnar stromatolites are based on flat-pebble conglomerate limestones, and the intervals of stromatolites are filled with thrombolites and a certain amount of ooids, occasionally earthy yellow micrites (Figs 4f and 6c). *Girvanella* clumps and biodetritus such as trilobites could be found in dark laminae. In addition, stromatolites are usually wrapped in thrombolites that mainly developed in subtidal zones with deeper water bodies. Therefore, it is revealed that irregular small columnar stromatolites grow in the upper part of subtidal zone with deeper water (Fig 7).

### 4.5. Stromatolites that surrounding flat-pebble conglomerate limestones

Stromatolites that surrounding flat-pebble conglomerate limestones mainly form in marls or flat-pebble limestones. Stromatolites grow around flat-pebble gravels, and the macroscopic characteristics are obvious. The flat-pebble gravels containing different sizes and roundness are found in surrounding rocks (Fig 5c). The phenomenon indicates that the flat-pebble gravels are suspended by the strong hydrodynamic force, but the strong hydrodynamic force is not enough to break them. Thus, it suggests that they primarily occur in middle-lower part of intertidal zone with higher hydrodynamic conditions.

To sum up, stromatolites mainly develop in the tidal flat environment, are relatively concentrated in the intertidal zone, and a small amount are distributed in the supratidal zone and subtidal zone (Fig 7). The type of stromatolites changes from simple to complex with the enhancement of hydrodynamic conditions.

## 5. Discussion

### 5.1. Biological factors

Although cyanobacteria play an important role in the construction of Precambrian stromatolites, it was not until the Paleozoic at the turn of Precambrian and Cambrian that intense cyanobacterial calcification episode appeared [28]. As far as the Cambrian is concerned, the relationship between microorganisms of constructed stromatolites and metazoan is relatively complex [29]. They have a very important influence on the growth and evolution of stromatolites.

Many bioturbated and bioclastic layers in the early Cambrian stratum in western Henan. However, there are no obviously bioturbated traces in stromatolitic limestones, indicating that stromatolitic growth process is less disturbed by metazoans. Five stromatolitic layers are topped by bioclastic stagnant layers, and the “trade-off” relationship between microbialites and biodetritus and trace fossils. It is proved that the disappearance of stromatolites is caused by the living space of stromatolites are occupied by the foraging and destruction of metazoans.

The microstructures of stromatolites are related to the composition and types of microbial mat [20]. The thinner dark laminae that are clearly demarcated from light laminae will form when there are fewer *Girvanella* and growth in the prostrate and horizontal manners (Fig 6a, 6b). The thicker and continuous dark laminae will be formed when *Girvanella* are flourished and growth in overlapping manners (Fig 4c, 4d). Briefly speaking, the thickness and morphology of stromatolitic laminae are affected and controlled by the flourished degree and arranged patterns of *Girvanella*.

### 5.2. Terrigenous materials

The growth or direct extinction of stromatolites is caused by the massive injection of terrigenous materials [20,30]. Small columnar stromatolites are often interrupted by thin-bedded mudstones, then fused to form smooth wavy stromatolites, which demonstrates that the morphological types of stromatolites are controlled by injection of terrigenous materials. At the same time, the top of the multi-layer stromatolites are mudstones, and there is no stromatolitic development in a certain range after the appearance of mudstones (Fig 2), which authenticate that input of terrigenous materials may lead to the extinction of stromatolites. The growth space of stromatolites is occupied and the seawater physical and chemical conditions become change due to the input of substantial terrigenous materials, which result in the disappearance of stromatolites. Thus, the growth and morphologies of stromatolites are influenced by terrigenous materials.

### 5.3. Hydrodynamic conditions

Based on the description and analysis of macroscopic morphologies of stromatolites, it is found that different types of stromatolites are caused by different hydrodynamic conditions. There are no sediments with stronger hydrodynamic conditions in the surrounding rocks of smooth wavy stromatolites (Fig 3c). However, the sediments such as oncolites and biodetritus with higher degree of fragmentation are found in the surrounding rocks of overlying hemispheric stromatolites (Fig 3b), indicating that the changes of the types of stromatolites correspondingly with the strengthening of hydrodynamic conditions. In addition, small columnar stromatolites form in weaker hydrodynamic conditions are often interrupted by thin-bedded mudstones, and gradually enlarged upward to form smooth-wavy stromatolites.

The research of microbial mats of modern stromatolites also declares that waves with different energy affect their growth by carrying particles to bury macroscopic algal and microbial mats [30]. The laminar characteristics of stromatolites and oncolites, biodetritus and other sedimentary structures in surrounding rocks reveal that hydrodynamic condition effects on morphologies of stromatolites. All in all, hydrodynamic conditions are primary factors that control morphologies of stromatolites.

### 5.4. Sedimentary substrates

The columnar and ridged stromatolites grow on hard substrates [30], which provide possible space for upward growth of stromatolites, while the soft substrates require that the stromatolites extend in the horizontal direction, such as algal laminae and smooth wavy stromatolites. Stromatolites that mostly develop on hard substrates present irregular small columnar and hemispheric (Figs 3b and 5b), while stromatolites that mainly grow on soft substrate show small columnar and smooth wavy (Figs 3c and 5a). Therefore, it can be seen that different sedimentary environments such as hydrodynamic conditions and water depth will form various sedimentary substrates and corresponding microbial communities, resulting in the formation of morphologies of stromatolites.

## 6. Conclusion

(1) The small plexiform, hemispherical-smooth wavy, small columnar, irregular small columnar stromatolites, and stromatolites that surrounding flat-pebble conglomerate limestones are recognized in western Henan, North China Carton, and they own different macromorphology and micromorphology. A large number of *Girvanella* fossils are preserved in dark laminae, which are mostly distributed in the prostrate or horizontal and winding or overlapping patterns, and form dark laminae of different thickness by interacting with inorganic calcium carbonate precipitation.
(2) The stromatolites principally develop in the intertidal zone, with a small amount are distributed in the lower part of the supratidal zone and subtidal zone, in which small plexiform and small columnar stromatolites occur in supratidal zone, hemispheric-smooth wavy, small columnar stromatolites, as well as stromatolites that surrounding flat-pebble conglomerate limestones primarily develop in intertidal zone, while irregular small columnar stromatolites are only distributed in the subtidal zone.
(3) The comprehensive research unravels that the micromorphology characteristics of stromatolites are affected by microorganisms and metazoan, the macromorphology characteristics of stromatolites are affected and controlled by terrigenous materials, hydrodynamic conditions and sedimentary substrates. The higher variation of hydrodynamic conditions, the more types of stromatolites. Moreover, the environment with more prosperous microorganisms and less terrigenous materials is more conducive to the development of stromatolites.
